# Subcutaneous acupuncture improves acyclovir-ineffective cephalofacial herpes zoster: A case report

**DOI:** 10.1097/MD.0000000000042066

**Published:** 2025-03-28

**Authors:** Zhenzhen Wang, Zhenming Zeng, Xiahai Zheng, Haiwei Gao, Haoxiong Chen, Yujia Chen

**Affiliations:** a Guangzhou University of Chinese Medicine, Guangzhou, China; b The Third Affiliated Hospital of Guangzhou University of Chinese Medicine, Guangzhou, China.

**Keywords:** case report, fire acupuncture, herpes zoster, subcutaneous acupuncture

## Abstract

**Rationale:**

Herpes zoster is a common skin disease that manifests as clustered herpes, postherpetic neuralgia (PHN), and other herpes zoster-related complications, as well as eye diseases and various visceral diseases. Pharmaceutical therapy may cause adverse responses and has limited efficacy. Therefore, exploring alternative and effective therapies is important for clinical physicians.

**Patient concerns:**

The patient was a 62-year-old female. She suffered from clustered herpes in her left forehead and head, which caused severe pain. The patient had received pharmaceutical therapy but did not respond to it.

**Diagnoses:**

Cephalofacial herpes zoster.

**Interventions:**

The patient underwent a 1-week course (4 sessions) of subcutaneous acupuncture. The treatment involved fire acupuncture (swift pricking) and common acupuncture (transverse insertion), both of which were performed on the superficial skin layer.

**Outcomes:**

After the 4 treatment sessions, the patient’s herpes disappeared quickly, and the pain was relieved. After the 12-month follow-up, the patient had no PHN.

**Lessons:**

Subcutaneous acupuncture improved the skin lesions condition of herpes zoster rapidly, and relieved the neuralgia effectively. It may prevent the PHN. For patients who are nonresponsive to antiviral medications, especially the elderly population at a high risk of developing PHN, subcutaneous acupuncture (fire acupuncture + common acupuncture) can be considered.

## 1. Introduction

Herpes zoster is a common skin disease caused by reactivation of the varicella-zoster virus, which was previously dormant in the sensory ganglia.^[1]^ Clinical symptoms manifest as clustered herpes along unilateral peripheral nerves and they commonly cause postherpetic neuralgia (PHN) and other herpes zoster-related complications as well as eye diseases and various visceral diseases.^[2]^ Antiviral drugs, such as acyclovir are frequently used to shorten the course of treatment. Because of the high risk of developing PHN, infected people usually need analgesic medication to alleviate pain and antidepressants to ease the negative emotions generated by pain. However, pharmaceutical therapy may cause varied degrees of adverse responses and has limited efficacy. Therefore, exploring alternate and effective therapies is important for clinical physicians. Here, we present the case of a patient with acyclovir-unresponsive herpes zoster of the head and face who appeared to benefit from subcutaneous acupuncture treatment.

## 2. Case report

**Figure 1.**
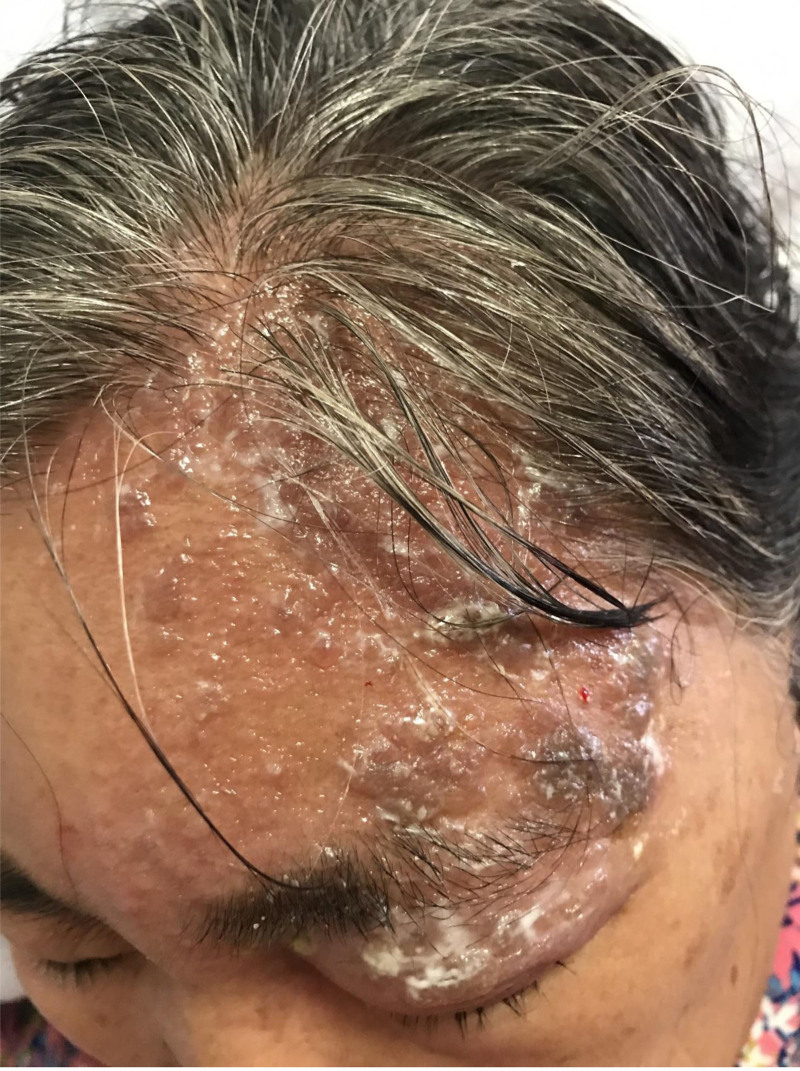
The herpes condition of the patient before acupuncture treatment.

A 62-year-old Chinese female patient visited the Third Affiliated Hospital of Guangzhou University of Chinese Medicine on June 23, 2023. After experiencing a burning sensation on her left forehead and head, she developed herpes on the 2nd day, causing severe pain, which worsened on the 3rd day. The patient visited the dermatology department and was diagnosed with herpes zoster infection. After 1 week of acyclovir (oral and topical) and pain medication, neither clustered herpes nor neuralgia decreased (Fig. 1). The patient discontinued medication and went to the rehabilitation department for acupuncture treatment.

## 3. Acupuncture treatment

**Figure 2.**
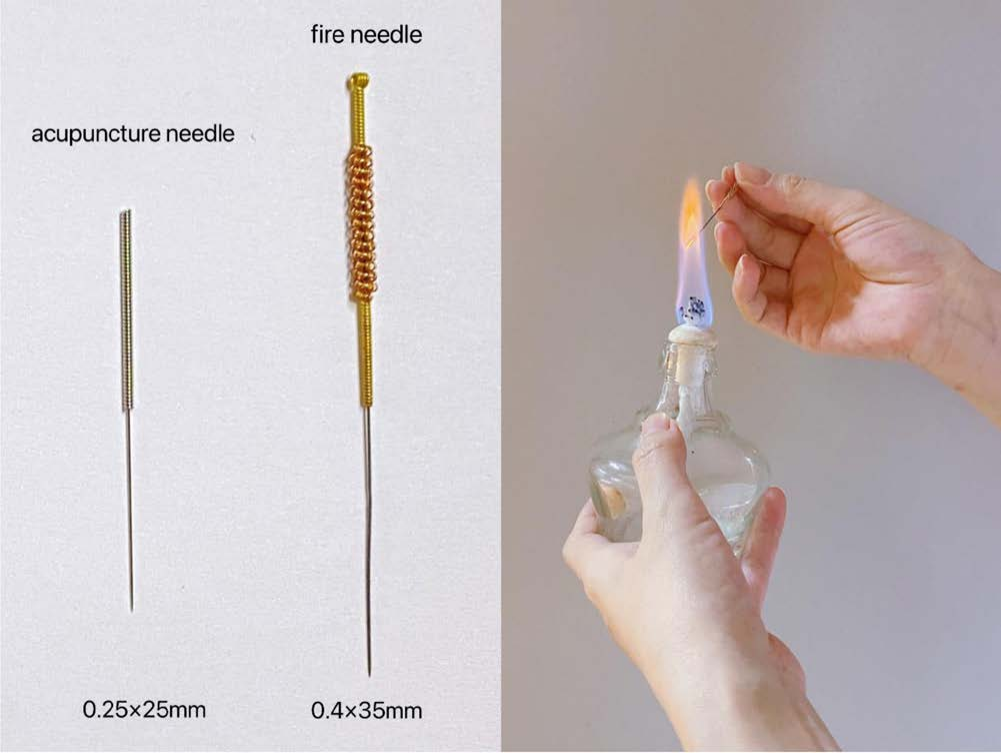
Illustration of common acupuncture needle and fire needle.

**Figure 3.**
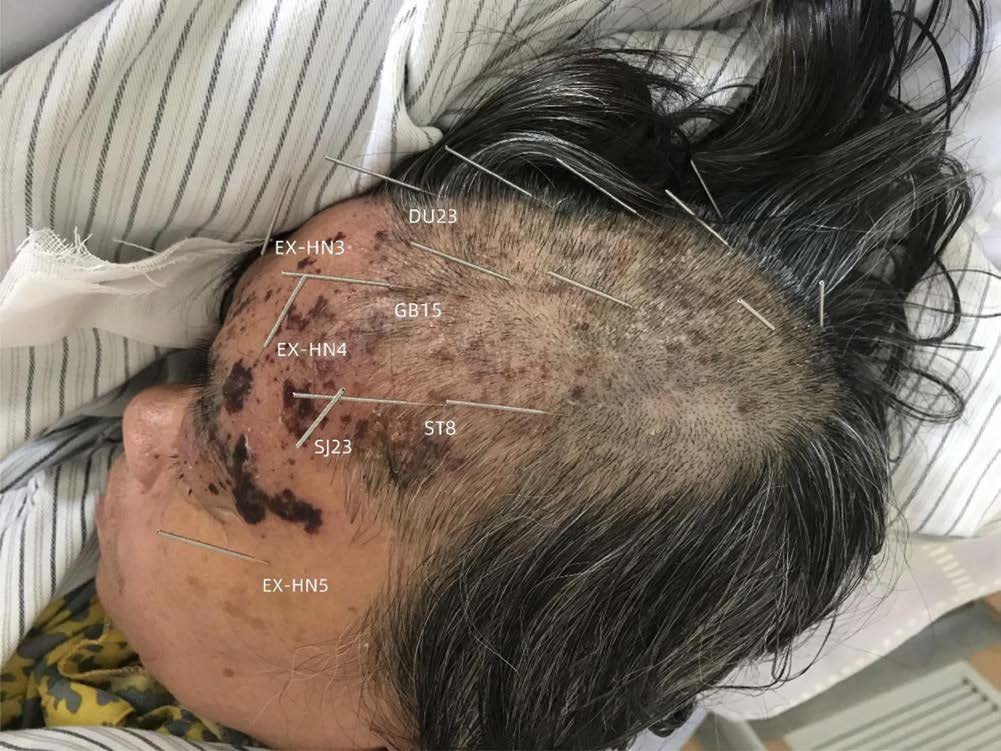
Acupuncture treatment and specific acupuncture point location.

The patient underwent a subcutaneous needling treatment regimen utilizing fire needles and common needles (also known as filiform needle) (Fig. 2), administered by an experienced acupuncturist. The treatment involved fire acupuncture (FA; swift pricking) and common acupuncture (transverse insertion), both of which were performed on the superficial skin layer. First, burn the sterilized fire needle (size: 0.40 × 35 mm; Zhenjiang New Area Great Wall Medical Supplies Factory, China) with an alcohol lamp until it turns red and bright (heat for about 8–10 s), then quickly prick the needle into the herpetic lesions to a depth of approximately 1 to 2 mm and pull out immediately. Each herpes site was pricked 2 to 3 times without retaining needle. Then, use sterilized disposable stainless steel acupuncture needle (size: 0.25 × 25 mm; Suzhou Acupuncture and Moxibustion Supplies Co., Ltd., China) to obliquely stab into the skin or the scalp. Choose acupoints based on the meridians in which the herpes is found. Figure 3 shows the specific position of the acupuncture: the left Taiyang (EX-HN5), Sizhukong (SJ23, needle tip reach to Sizhukong), Yuyao (EX-HN4, needle tip reach to Yuyao), Yintang (EX-HN3, needle tip reach to Yintang), Shangxing (DU23), and Toulinqi (GB15) are inserted transversely along the meridians (respectively along the governor vessel and gallbladder meridian) to the top of the head. Touwei (ST8) is inserted transversely from the head to the rear of the head. The needle points may be added appropriately based on the herpes distribution, and needles were inserted transversely every 3 cm to a depth of 20 mm. The selection of acupuncture points may deviate from the acupoints, but try not to deviate from the meridians. The needles were retained in the points for 30 minutes each session. The patient received treatment every other day for a total of 4 sessions. No drugs were used throughout the treatment.

## 4. Outcome

**Figure 4.**
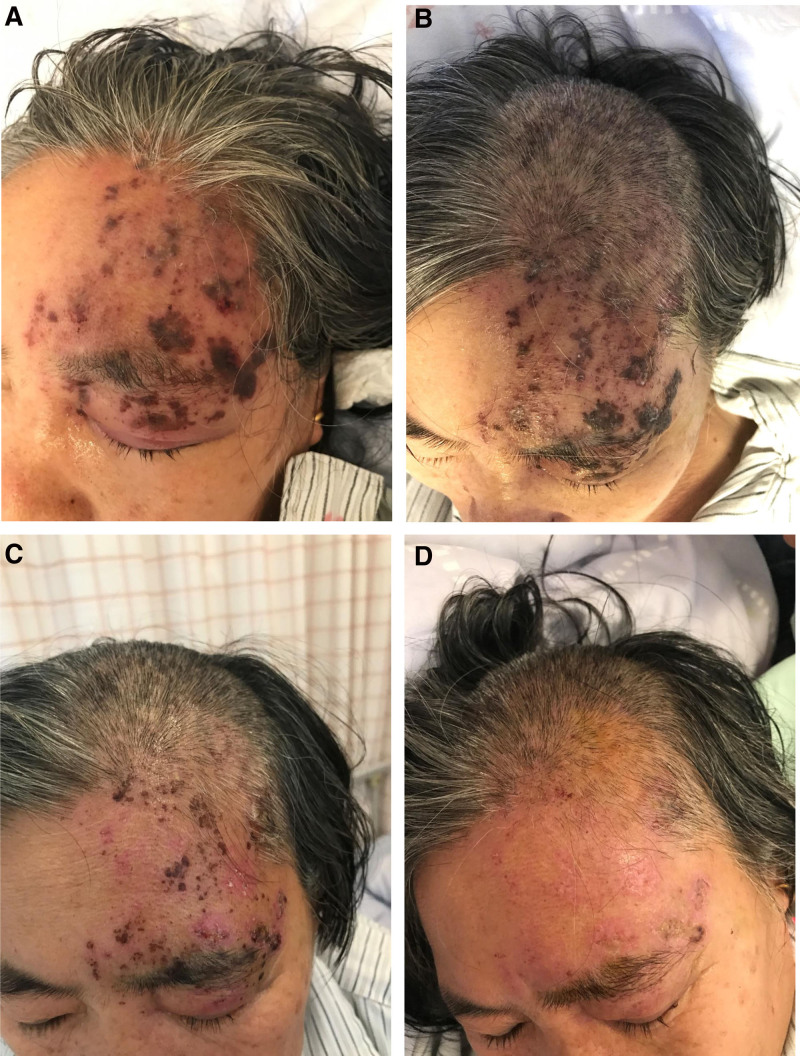
Changes in patient’s skin herps: (A) 1 day after treatment initiation (post-1st session), (B) 3 days after treatment initiation (post-2nd session), (C) 5 days after treatment initiation (post-3rd session), (D) 7 days after treatment initiation (post-4th session).

**Figure 5.**
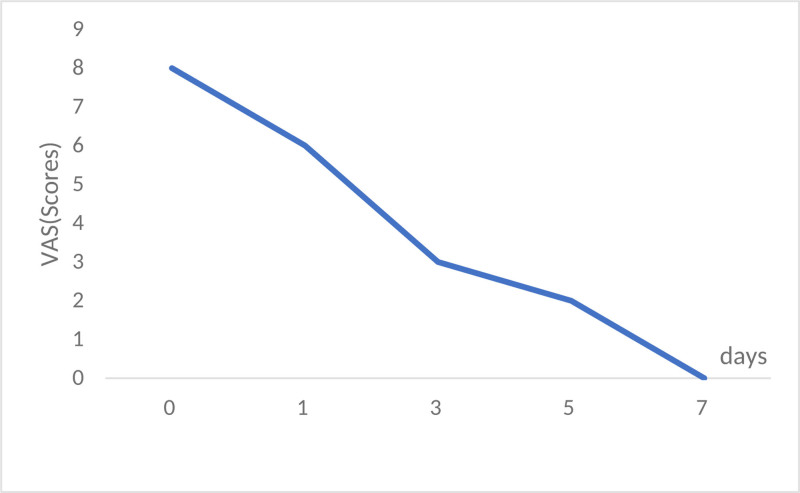
VAS scores along the course of the disease. VAS = visual analogue scale.

After the 1st treatment, the number of herpes was significantly reduced (Fig. 4), pain was significantly relieved (Fig. 5), and sleep improved. After the 3rd treatment, the patient’s herpes was mostly gone, with only occasional slight pain. After the 4th treatment, the patient’s frontal skin was smooth, there were no new herpes, and the pain basically disappeared. The patient’s recovery speed is fast, and the course of the disease is obviously shortened. After 12 months of follow-up, the patient remained free of postherpetic neuralgia or other sequelae.

## 5. Comment

PHN is a debilitating sequela that profoundly impacts patients’ quality of life, particularly in older adults. Mallick-Searle et al^[3]^ found that the incidence of PHN increases with age, making older age a significant risk factor for this condition. Therefore, it is clinically important to explore prevention methods for PHN after herpes zoster infection in the older population. In the early stages of shingles, early intervention to mitigate PHN risk is crucial. Current routine antiviral therapies cannot consistently and reliably prevent or alter the onset of PHN, and they are only partially effective in slowing disease progression during the acute phase.^[4]^ Currently, the mechanism of PHN is not yet clear, and some studies have found that severe acute pain and extensive skin lesions during herpes zoster may predispose patients to PHN,^[5]^ highlighting the need for interventions to accelerate skin healing and reduce neural damage.

In this case, despite a standard course of acyclovir therapy, the patient demonstrated persistent herpetic lesions and neuropathic pain with inadequate therapeutic response. Subsequent implementation of subcutaneous acupuncture resulted in rapid resolution of both herpetic lesions and neuropathic pain within a shorter timeframe (Figs. 1 and 4). The observed temporal association between acupuncture intervention and clinical improvement—particularly the accelerated symptom resolution following failed antiviral therapy—suggests a potential therapeutic effect beyond natural course, though causal inference remains limited in this single-case observation. After the 12-month follow-up, it was found that the elderly woman had no obvious sequelae. We speculate that the prevention of PHN in this case is due to the effect of acupuncture intervention, which can shorten the course of disease and rapidly reduce the area of skin lesions.^[5]^

Mechanically, evidence indicates that FA suppresses neuroinflammation by downregulating pro-inflammatory mediators, including prostaglandin E2, substance P, and tumor necrosis factor-α, thereby alleviating pain hypersensitivity.^[6]^ Additionally, acupuncture modulates the expression of nociceptive signaling molecules such as interleukin-6 and calcitonin gene-related peptide, which reduces pain sensitization and restores normal pain thresholds.^[7]^ Clinically, we observed that FA can cause herpes to dry out faster and skin lesions to heal faster. In this case, we choose subcutaneous acupuncture that stimulates the subcutaneous tissue of the skin lesion, promote subcutaneous tissue healing, reduce inflammation, and prevent nerve damage. Furthermore, we believe that transverse insertion has a greater amount of stimulation under the skin or the scalp than perpendicular insertion, which may better motivate lesion tissue repair and promote disease recovery.

While this outcome suggests that subcutaneous acupuncture (fire acupuncture + common acupuncture) might help shorten acute herpes zoster progression and potentially reduce PHN risk in medication-resistant elderly patients, controlled trials are required to confirm whether the observed benefits specifically stem from the intervention rather than natural course. Future studies should prioritize comparing subcutaneous acupuncture with standard therapies in high-risk PHN populations.

## Acknowledgments

The authors would like to thank the patient for agreeing to publication of this report.

## Author contributions

**Conceptualization:** Zhenzhen Wang, Zhenming Zeng.

**Data curation:** Xiahai Zheng.

**Resources:** Haoxiong Chen.

**Supervision:** Zhenzhen Wang.

**Validation:** Yujia Chen.

**Writing – original draft:** Zhenming Zeng, Haiwei Gao.

**Writing – review & editing:** Zhenzhen Wang.
